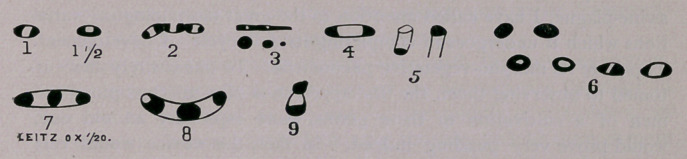# The Corn-Stalk Disease in Cattle

**Published:** 1889-08

**Authors:** Frank S. Billings

**Affiliations:** Director of the Patho-Biological Laboratory of the University of Nebraska


Buffalo Medical and Surgical Journal

VOL. XXIX. AUGUST, 1889. No. 1.

### Original Communications.

### *THE CORN-STALK DISEASE IN CATTLE.*

BY FRANK S. BILLINGS,

Director of the Patho-Biological Laboratory of the University of Nebraska.

(*Continued from July number.*)

IMMEDIATELY upon discovering the close resemblance of this germ to that of the Southern cattle plague, and before my cultures had had time to develop, or I had tested its malignity on any animals, I wrote to Mr. R. M. Allen, manager for the cattle company named, making inquiries as to the possibilitiy of its having come in contact with any feed that could have in any way been in contact with material polluted by Texas cattle during the previous Summer or Fall months, and received the following reply, which is inserted on account of its historical connection with these investigations:

"AMES, Neb., Jan. 11, 1888.

"*Dear Sir*—I cannot see any possible chance of the infection of the steer from hay polluted by Texans. The steer was from the J. E. Boyd herd on the Cheyenne river, Wyoming territory, and I do not know of any Texas cattle going into that country the past Summer. We have Texas cattle of our own here, but they have all passed a Winter in Wyoming. There have been no hogs about the cattle in any way. I am sorry I failed to examine another steer that died in the lot—thought it was constipated [a condition common in the corn-stalk disease—B.], but I think it died from a different cause. We lose a number of steers that die suddenly, but in such cases we generally find a diseased liver [the liver is badly diseased in the corn-stalk disease—B.], and other appearances of excessively rich and concentrated feeding. We did not know that the steer was sick until we found it dead. We had examined this lot particularly for sick cattle, having recently lost one out of the same pen."

Here, then, was a new disease discovered, and one belonging to that group of extra-organismal (etiologically) septicemiæ, and caused by still another member of the ovoid-belted class of bacilli, or germs; but where the organism came from, or how the disease originated, was still a perfect mystery, which was not much cleared up by the appearance of a second outbreak in an entirely different part of the State, from which I also received material and the following communications.

### THE CORN-STALK DISEASE AT CORTLAND, NEB., MARCH, 1888.

The material from this outbreak came to me in a still more satisfactory manner than that previously mentioned. It seems it was originally sent to the State Live-Stock Commission, but as neither they nor their State Veterinarian were competent to make any use of it, it was sent to this laboratory, and had it not had the name of "Dr. W. S. Brayton, Beatrice," on the wrapper, it would have been impossible to trace the matter any further, for the sublime simplicity and ignorance of the State's watchful guardians of our live-stock interests, placed them entirely beyond the pale of either useful knowledge or practical information.

Microscopical examinations of the fresh organs revealed the presence of apparently the same organism as had been found in the Ames case, and the inoculation of small animals enabled me to obtain pure cultures, by which its identity with that germ was sufficiently demonstrated. Upon writing to Dr. Brayton, I was favored with the following polite reply:

"BEATRICE, Neb., March 20, 1888.

"F. S. BILLINGS, Lincoln, Neb.:

"*Dear Sir*—Yours of March 18th received. The history of the cattle, as near as I can find out, is as follows: They were shipped from Osage, Iowa, about September 1, 1887, to Cortland, Neb. When starting from Osage, they were in apparent good health, and at Cortland, were unloaded and given in the charge of a Mr. ——— to winter. The cattle were herded on high ground, getting their water from a small creek which runs through the same. As soon as cold weather commenced, they were taken about five or six miles from Cortland, and put into a lot containing somewhere from five to six acres on a creek bottom, and were allowed to run to flax-straw and oat-straw for feed, and getting their water from the creek which ran through the lot; I think the same one that runs through the pasture in which they were herded during the Fall, and in which they are at present. . . . In this lot was some timber (I do not know how much), which was their only protection from the weather. From this lot they were moved to their present location, and allowed to run to a millet stack and to some oat-straw stacks. On March 13th, I held post mortems upon two dead animals—one a cow (in calf) and a yearling steer. The cow had died the night before, and was in a fair condition as regards flesh, but the steer was poor. I found on post mortem, first, that the blood was of an unnatural color and seemed to be thick. The heart had a blood-clot in each side, and the posterior aorta contained a clot for about a foot from the heart. I found no lesions in the mouth, but when the stomach was reached, the lining membrane of the rumen would peal off in large patches. The discharge from the bowels was a little thinner than natural and streaked with blood. The lungs seemed in a healthy condition. The piece of lung sent was from the lung on the under side, which I think caused it to be so congested. The liver was about its natural size. The spleen about natural, with the exception that it looked blacker than usual.

"The history is as follows: These cattle would be all right at night, but in the morning there would be one or two that could not get up, but showed no signs of pain. Some of these would get up with help for once or twice, and then die in the course of three or four days, and others would not get up at all, and die in from twelve to twenty-four hours. . . . The trouble seems to affect the cattle in the best condition. Cows abort their calves, and seem to do well after it. There are no external appearances of disease. The cattle have had no shelter this Winter, except the timber already spoken of. I saw both hogs and horses among the cattle, but was informed that there had been none of them sick. I saw some of the hogs eating a portion of a carcass of one of the cows."

Though I had again discovered the same micro-etiological organism that I found in the material from the steer of the Standard Cattle Company at Ames, Neb., a few weeks previously, and proved its malignancy in this case also, still I was completely in the dark as to its source, or origin—that is, in what manner the cattle got at it. That the disease was due to feeding on food polluted with the germ, I felt fully convinced. I had no data up to this time pointing to corn-fodder as the cause—in fact, I doubted the whole business, though really knowing nothing about it, save that it seemed improbable that dry fodder alone, or even smut, could possibly cause it. It will be seen that Dr. Brayton does not mention corn-fodder, or stalk-fields, in his letter, and at the time I did not know enough to be suspicious and ask any questions in that direction, but he does mention millet and hay-fodder, which opens up the question, Can or does the germ invade these materials also? An answer to this can only be given by practical experience and observations with the assistance of the scientific botanist and the pathological investigator.

In order to show the value of scientific investigation, even when the ultimate result sought for has not been attained, let me say that all these experiences clearly taught me that the specific cause was in some way connected with the food the cattle were getting; so I advised Dr. Brayton to change the same entirely, which was done with the most happy results.

### CORN-STALK DISEASE AT FREMONT, NEB., FEBRUARY, 1889.

By referring to an earlier part of this report, the reader will see that "Dr. Osborn, State Veterinarian" (of Nebraska), visited this outbreak, and on February 7th said: "There is no contagious disease known to veterinarians which affects the third stomach of the cow, and that was the seat of the disease in every case that I examined. The third stomach, or manifold, was packed with dry food, which, taken in the fingers, crumbled like flour."

The above shows a terrible lack of necroscopical ability, for, in the first place, the condition of the stomach named is, in a varying degree, common to every acute infectious disease in cattle, accompanied by an excessive rise of temperature; and again, as will be soon shown, there were essential, specific, and pathognomic lesions in these animals of just such a disease, which the veriest tyro should not have allowed to escape his notice.

No sooner did I see the reports of this outbreak in the daily papers, than the suspicion arose that it was probably the same disease from which I had previously examined material and procured the same etiological organism from, at Ames and Cortland. Unfortunately, a most peremptory engagement—the farcical investigation ordered by the Legislature—rendered a personal inspection of the outbreak entirely out of the question. Hence, I dispatched the most trustworthy veterinarian at my command, though not such as I would desire as an expert in necroscopical observation, to visit the outbreak and bring me back material in sterilized bottles with which he was supplied, and which he knew how to collect in a suitable manner. As said above, I could not expect very detailed reports of the gross pathological lesions, still Dr. Thomas's very brief report of what he did see is sufficient to show that much more serious lesions were present than those reported by Dr. Osborn—lesions which directly point to a malignant infectious disease, which interfered most seriously with the circulation, and which must, of necessity, have been accompanied by an excessive rise in temperature. The animal examined by Dr. Thomas was killed by him, and immediately opened, the material being at once placed in the bottles previously mentioned.

His report is as follows:

"Symptoms and post mortem conditions of Mr. John Delaney's cattle, also how they have been cared for since December 1, 1888: Lost fifty-two head up to February 7th (1889); commenced dying five or six weeks previously. Mr. Delaney's herd was composed of 170 head, divided into three lots, viz., 100 cows and heifers, forty-six last year's calves, and twenty-four fat steers. Deaths have occurred as follows: Seven fat steers, eight or nine calves, and thirty-seven cows and heifers.

"Mr. Delaney's herd is in good condition, though a few are thin in flesh. The 100 head have been fed hay, and run in the stalk-fields days. The fat steers have been fed corn and hay. The calves were fed millet until February 4th. They also had some corn. The entire herd drinking from one tank supplied by a wind-mill—all having a certain amount of salt. The hay fed is in very good condition; the cows also drinking from the Elkhorn river when running in the field. The first symptoms noticed are switching the tail, some of them shivering, followed by bellowing, staring eyes, chasing pigs and chickens, in fact almost anything coming in their way; straining so violently that many of them evert the rectum, and evacuate only a small amount of feces, somewhat covered with mucus. They become lame, paralysed, and usually stand until a short time before death. Strongest ones live longest, and the wildest die in the shortest time. They are sick from eight hours to seven days. The majority of them become wild or delirious, and die within twenty-four hours after the first symptoms are noticed. Fifteen to twenty were shot to prevent them doing damage. Five or six did not get delirious.

AUTOPSY.

"Pleuro-pneumonia sufficient to kill; the entire lung being congested and the lower portions of the lobes solid; inflammation of the pleura, and about two gallons of serum in the thoracic cavity; stomach all in good condition; liver very firm and pale-colored; gall bladder well filled; urinary bladder filled; intestines inflamed.

"Mr. Delaney stated that, in those he examined, the liver looked 'half-cooked or white, and the gall bladder as large as a hog's bladder blown up.' Others stated that, of those examined, the majority of the gall bladders were ruptured. In one case the rectum divided, black and gangrenous, also a portion of the small intestines the same.

"Mr. Delaney's farm is on the Elkhorn and Platte river bottoms, about five or six miles north-east of Fremont, a short distance from the Elkhorn river."

It will now be remembered that Dr. Osborn also said: "The hay and stalks are exceedingly dry, and the cattle not having sufficient quantities of water and salt, congests are found." Just what "congests" means pathologically, is more than I can explain. Dr. Thomas was especially directed to look up the water question, and it is to be seen that in no way could the cattle have been without a sufficient supply of water to answer the requirements of nature.

The material brought in by Dr. Thomas consisted of fresh blood from the heart, which was coagulated, the coagulum being solid and of a dark purple-red color; serum over it straw-colored and clear.

There were pieces of the organs in a tin box, and they were somewhat frozen; for, according to my orders, they were to be allowed to freeze at once, and kept so immediately after being removed from the animal.

*Lung.*—The pleura covering the piece of lung was much thickened, presenting an irregular, shreddy surface of a yellowish-red color, interrupted by numerous small red centers; the vessels of the interlobular spaces were engorged, much resembling the condition frequently seen in acute pneumonia in swine-plague; the lobuli were solidified, some being of greyish-red color, others purplish grey-red, while still others were of a yellowish grey-red color and very anemic; the cut surface was exceedingly edematous; interlobular tissue swollen; many lobuli presented centers of a diffuse dark purple-red color, between which were others of a pearly-grey color, others dull grey, and still others yellowish-grey, with a varying amount of reddish tissue between them; bronchial tubes filled with straw-colored, coagulated material. As mentioned previously, such a description would answer equally well for a form of pneumonia met with in swine-plague, especially in eight or ten-day cases; in fact, the structure of the lung having such a color resemblance in cattle and swine, there is no pathologist living who could have told this piece of lung from that taken from a similar case of swine-plague, and the conviction would have been still more strengthened by the examination of covering glass specimens of the tissues and the blood of this animal; but the fact that swine are known to be insusceptible to this disease, entirely shuts out that probability. The closeness of the mere microscopical resemblance of this organism to that of swine-plague, is very well illustrated by the accompanying letter from Prof. T. J. Burrill, the most accomplished mycologist in this country, to whom I sent cultures and a slide:

"CHAMPAIGN, Ill., March 9, 1889.

"*My Dear Doctor*—Yours of the 3d inst. reached me yesterday, apparently after some delay *en route*, and the box came this morning safe and in good order. The tubes are all fertile, and, as far as examined, have pure cultures. *I have not fully studied the microbe, but am not a little surprised that the thing is so near like hog-cholera in its microscopical characteristics.* But I find no difficulty in applying your description. I have already tried inoculation in a rabbit, and will further study your cultures and let you know result. I have not, but will also look up my old slides, and compare. Will write you early next week. In the meantime, I congratulate you upon the progress already made in this entirely new work. Bravo!

"Hurriedly but truly yours,

"T. J. BURRILL."

To return to our pieces of organs:

*Liver.*—Capsule normal, cut surface very opaque, excessively swollen and anemic, and of a dull, greyish, brown-red color; peripheries of the acini of yellowish-grey color. Center reddish.

*Kidneys.*—Cortex much swollen, anemic, opaque, yellowish grey-red in color; vasa and tubuli recti much distended; medullary substance bright red, interrupted by a very pregnant distension of the tubes, and an occasional large blood-vessel. A small piece of the small intestine presented a very much swollen mucosa, covered with a thick viscid coating, and of a diffuse yellowish-red color.

*Covering* glass specimens from all these organs gave, apparently, pure conditions of one and the same organism, corresponding exactly to those found in the Ames and Cortland outbreaks. A hanging-drop, prepared directly from the blood coagulum, at once shows the organism to possess most active movements, corresponding exactly to those of the swine-plague and Southern cattle-plague organisms, and possessing the same manner of development.

MORPHO-BIOLOGICAL CHARACTERISTICS OF THE GERMS OF THE SOUTHERN CATTLE-PLAGUE, THE AMERICAN SWINE-PLAGUE, AND THE CORN-STALK DISEASE IN CATTLE.

These microörganisms are neither to be classed with micrococci or bacilli, though some class them with the latter. They are not round objects like the former, or regular rods like the latter. They belong to that intermediate group to which, for convenience sake, patho-bacteriologists are beginning to give the name "bacteria," which, while not perhaps a scientific classification, has many practical reasons in its favor. Their longitudinal diameter is about twice that of their transverse. They are ovoid. Their ends are rounded. If an endeavor be made to differentiate these germs from one another by a microscopical examination, we shall find it impossible. They are approximately of the same size and shape. Fresh specimens of either will not differ so much in dimensions—under the microscope—as old cultures of either will from fresh ones, or different individuals in the same old cultures. They are about one-sixth the transverse diameter of a red blood-cell in length. In one way, however, they can be easily differentiated by microscopic examination.

The swine-plague germ has a far sharper chemical affinity(its poles) for the blue and violet tinctions than that of the southern cattle-plague or the corn-stalk disease, and the latter possesses a special affinity for fuchsin, while the former does not. The swine-plague organism is also more bacillary than the others.

Whatever the tinction used, if applied *lege artis*, the ends, or poles, of these microörganisms will show a greater specific affinity for the coloring material; while the middle portion of the body has far less, unless the exposure to the tinction is unduly or longer pushed, when this portion of the body will eventually color. The capsule of these germs seems to be composed of the same material as the ends, as it colors in the same manner, thus presenting a delicate line of substance connecting the two-colored, coccoid, ends or poles. The most practical illustration which can be given of the microscopic appearance of these organisms is to take a small white bean and paint both of its ends and two of its sides blue or red, leaving the middle portion unpainted. Looking down upon such a bean would give the observer an almost exact picture of these microörganisms.

Like the genuine and only germ of the American swine-plague, the microörganisms of the southern cattle-plague and corn-stalk disease are motile in fluid-cultivating media when observed microscopically, as well as in the blood-serum of diseased animals.

The movement of these germs is not the active shooting or straight-out movement of many organisms, but they change their locality in the field, they turn over and over, rise and fall in the drop, move sideways, and when two are united together twist in various directions, in their endeavors to separate, just as we try to break a rather tough stick in two.

In my earlier descriptions of the microörganism of the American swine-plague, I have called attention to the great morphological variations which it undergoes in completing its full cycle of development. These are its morpho-vegetative phenomena. To one entirely unaccustomed to observing them, the first appearance of a microscopic specimen of a cultivation of these germs, more especially an old one, would prove very puzzling indeed. In fact, the novice would very often conclude that his cultures had become polluted by micrococci, so plentifully are these objects apparently represented. They simply represent a vegetative or embryonal period in the development of micro-etiological organisms. The views of Hüppe, an eminent German authority, are very misleading upon this point. He describes, or classifies, this class of germ as "micrococci." It would be equally logical, however, to call an ovum a man, or an apple-seed an apple tree. It is far more practical for the patho-bacteriologists to stick to the name "cocci" for all round objects—not spores—which have equal diameters in their mature form, and which color diffusely, and to call these ovoid organisms bacteria, where the longitudinal diameter is not much more than double the transverse. As to bacilli, spirilli, etc., there need be no dispute, so plain are their morpho-characteristics.

The mature micro-etiological organism of the American swine- and southern cattle-plague, corn-stalk disease has been described above—Fig. 1—as resembling a white bean with its ends and sides so painted as to leave the middle portion of the body untouched, as we look down upon it. That is the picture which the eye of the observer generally receives; but a more exact inspection of a stained covering glass specimen will show that the above is not always the appearance presented to the eye, even by the mature germ. Many specimens may be seen in which the white belt does not extend entirely across the object, and there will be more uncolored substance upon one side than the other (Fig. 1½). At first I mistook this appearance for the accumulation of the uncolored substance in this way during the process of its secretion by the pole-ends, which I take to be the method by which this non-coloring material is produced. The whole organism is surrounded by a capsule, but naturally we do not see that portion covering the pole-ends, as it colors at the same time with them. The question now arises: If the whole capsule colors, why do we only see evidence of the same on the sides and not on the part presented uppermost to the eye, which appears uncolored?

Whether or not this appearance of more color in the capsule upon one side or the other is due to the action of the heat in drying the covering glasses, is more than I can say, but the reason that we only see the capsule colored on the sides under appropriate treatment, is very evident. It is an optical phenomenon.

The whole capsule colors exactly alike, with the above exception, but being so extremely delicate that we do not perceive the color in that portion presented to the eye by the middle of the object, on account of its thinness; but in looking at the side, we look through more material and hence see more color, just as in looking through a glass-slide or piece of window-glass, it appears clear; but if we look through more volume of glass, by looking at its edge, we perceive a more or less greenish shade, according to the quality of the glass.

Again we may see two or three of these organisms joined together, all presenting the normal characteristics of full maturity (Fig. 2). I have never seen more than three thus united, except in very old cultures. In general, they appear either singly or in pairs. In very old cultures, these microörganisms become thinner, more rod-like, and color more diffusely with the same degree of exposure to the tinction, and the white substance is either not visible at all or is very faint (Fig. 3). Again, such old cultures are very replete in apparent micrococci of various dimensions, which might lead one into the error of assuming that his cultures had become polluted. I call this last condition that of coccoid degeneration (Fig. 3); or we may see unusually long objects, the longitudinal diameter of which being twice or three times that of the mature germ, the white or uncolored substance occupying a corresponding extensive amount of space, while the refracting or colored pole-ends may be somewhat larger, or of the same size of those of the mature object. This condition represents the first step in the development of these organisms: that is, they become longer, and more of the white non-refracting material is secreted (Fig. 4).

The next step in the process of vegetative development is the separation of one of the pole or coccoid ends, which becomes free and for a moment is exactly round like a coccus; and, as in a hanging-drop culture (to which I always add a very slight amount of an aqueous coloring solution), one will naturally see a very large number of these coccoid objects, on account of the fact that each individual germ present is continually going through the same process of multiplication. Here, again, we may see a phenomenon that might be misleading: one of the coccoid ends having been separated, the other still remains attached to the white non-refracting material; and, as evidence that the refracting pole-ends have a greater degree of specific gravity, as well as chemical composition, we may see in the continual tumbling about and turning over and over of these objects, a white, round, or nearly so, colorless, non-refracting object, or numbers of the same. When the microörganisms in such a hanging-drop culture have died from want of nourishment, we may see a large number of these objects, which can easily be mistaken for spores. But if we inoculate a new hanging-drop culture from the same material used to prepare the former, it will be found impossible to fall into any such serious error. It will be easily seen, then, that these uncolored refracting points keep continually going out of sight, their place being taken by the non-refracting point still attached to the other end of the white substance. By watching one and the same organism in its continuous turning over and over, first one appearance and then the other will be presented to the eye, until the second end, coccoid, has become detached (Fig. 5).

What becomes of this colorless refracting middle piece?

I do not know.

To my mind, this material within the capsule which does not readily take up the tinction, is a fluid, and it seems to me as if this fluid became free with the separation of both pole-ends, and that the capsule underwent dissolution at the same time. That this white belt (in the complete organism) does not represent a spore condition or have any relation whatever to spores, is entirely beyond all question, as I have now searched diligently for spores for over a year in both old and new cultures of the swine-plague germ, and in others made at all kinds of temperature within the bio-limits of these organisms, but in vain.

These objects being so exceedingly minute, it takes some time to educate the eye so that one can perceive every phase of their development. There are days when one cannot study them continuously at all. The best way to study hanging-drop cultures, when one desires to spend several hours over them, is to first make some cover-glass specimens of the same material, or take slides of the same object and observe such for about half an hour, thus preparing the eye to see what one wants to see in the living developing organism. Unless this is done, some very essential points will be surely missed, and some preventable errors fallen into. With anything less than a power of 800 diameters, no one should attempt to study these organisms, and then only when aided by the best of Abbe condensers and oil-immersion lenses.

We left our studies with the mature object proliferated into its first distinct stage of vegetative differentiation. We had two coccoid objects before us; that is, two round objects, their diameters being the same in any direction. If colored, they color throughout: that is, diffusely. Were these objects to remain in this condition, they would be indeed micrococci. They do not, however. They almost immediately begin to increase in a longitudinal direction, but in this condition they still stain diffusely.

In my first description of the swine-plague germ, I said that the next biological phenomenon was the appearance of a delicate white line separating this ovoid object into two halves. The above, while not exactly an erroneous description, is certainly anticipated by another phenomenon in the evolutional development of this coccoid diffusely-coloring object into the mature germ of any of this class of diseases. That this white non-coloring substance is a secretion of the two pole, or coccoid, ends of these "belted" germs, is beyond all question, as well as that it has a different chemical composition. These two facts, when taken together with the previously stated one, that the white substance almost, if not instantly, disappears from view the moment both of the coccoid—pole—ends have become shed off, segmented, leads directly to the following hypothesis:

May not this white substance constitute, aside from the capsule, the ptomaine, or essential poisonous pathogenetic principle, in connection with these "belted" septicemic germs, and may not this process of the immediate dissolution of this white substance be the means by which this ptomaine gets into solution, and thus permeates the fluid-cultivating media and the blood?

To my mind, this supposition is worthy of consideration. The fact that we can find no evidence of the development of permanent spores by these germs, and that this white substance is a secretion of the pole-ends, goes largely to support this hypothesis.

The phenomenon above spoken of as anticipating the formation of the segmenting white line which separates the two darker portions of these organisms is, that this white substance first appears in the center of the body of the dense, dark, ovoid object as the minutest of white specks, which gradually increase in size and quantity, and extends across the entire object, the white line being at first broader in the middle but gradually widening until it completely and clearly separates the two pole (coccoid) ends, and the mature object is again presented to our view. (Fig. 6.)

We have thus described the normal or general cycle of development of the micro-etiological organisms of the American, English, and European swine-plagues, the American southern cattle-plague, hen cholera, the German "Wild seuche" (of deer, swine, and cattle), and rabbit septicemia, and the corn-stalk disease, all of which diseases are caused by a member of this class of "belted" germs, and should be classed as extra-organismal, local, or land septicemia. It seems to me that the germ of yellow fever, as well as the disease itself, should also come into this group[1].

[*To be concluded in September number.*]

1. See an article on the Germs of Yellow Fever lately published in *Medical Times and Register* of Philadelphia.

## Figures and Tables

**Figure f1:**